# Osteosarcoma Models: From Cell Lines to Zebrafish

**DOI:** 10.1155/2012/417271

**Published:** 2012-03-15

**Authors:** Alexander B. Mohseny, Pancras C. W. Hogendoorn, Anne-Marie Cleton-Jansen

**Affiliations:** Department of Pathology, Leiden University Medical Center, Leiden, P.O. Box 9500, L1-Q, 23000 RC Leiden, The Netherlands

## Abstract

High-grade osteosarcoma is an aggressive tumor most commonly affecting adolescents. The early age of onset might suggest genetic predisposition; however, the vast majority of the tumors are sporadic. Early onset, most often lack of a predisposing condition or lesion, only infrequent (<2%) prevalence of inheritance, extensive genomic instability, and a wide histological heterogeneity are just few factors to mention that make osteosarcoma difficult to study. Therefore, it is sensible to design and use models representative of the human disease. Here we summarize multiple osteosarcoma models established *in vitro* and *in vivo*, comment on their utilities, and highlight newest achievements, such as the use of zebrafish embryos. We conclude that to gain a better understanding of osteosarcoma, simplification of this extremely complex tumor is needed. Therefore, we parse the osteosarcoma problem into parts and propose adequate models to study them each separately. A better understanding of osteosarcoma provides opportunities for discovering and assaying novel effective treatment strategies.

*“Sometimes the model is more interesting than the original disease”*

PJ Hoedemaeker (1937–2007).

## 1. Introduction

Osteosarcoma is a collective name for a broad spectrum of osteogenic tumors [1, 2] of which the majority consists of high-grade primary sarcomas referred to as “conventional osteosarcoma.” Conventional osteosarcoma is characterized by high local aggressiveness and rapid metastasizing potential resulting in an early onset tumor (mean age is about 17 years) [3] with poor survival—less than 60% after surgery and high dose chemotherapy, less than 20% after relapse or chemoresistance, and less than 15% without chemotherapy [4–8]. Histologically a wide spectrum of conventional osteosarcoma is found; however, without clear significant differences of clinical outcome related to the subtype [9]. The histological heterogeneity of osteosarcoma might indicate a multipotent cell of origin, which is most probably the mesenchymal stem cell (MSC) along its path of differentiation to the osteoblastic lineage [10–12]. A good comprehension of osteosarcoma origin and etiology is complicated by several factors like its extreme rearranged genome, the lack of precursor lesions and the high genetic instability inhibiting the detection of the driver genes. Furthermore, most osteosarcoma cases occur sporadic—without obvious hereditary cause—with an early onset resulting into a full-blown tumor at the time of diagnosis. Moreover, although osteosarcoma is the most common primary bone tumor and in the pediatric age group it is the second highest cause of cancer-related death, its overall prevalence is low (4-5 per 1.000.000, world wide) [2], making it difficult to study the disease in large groups. Another limitation is caused by the administration of presurgery high-dose chemotherapy, which has proven its essence for patient's survival but which, when effective, eradicates all cancer cells leaving barely any viable tissue in the resected tumor to study.

**Figure 1:**
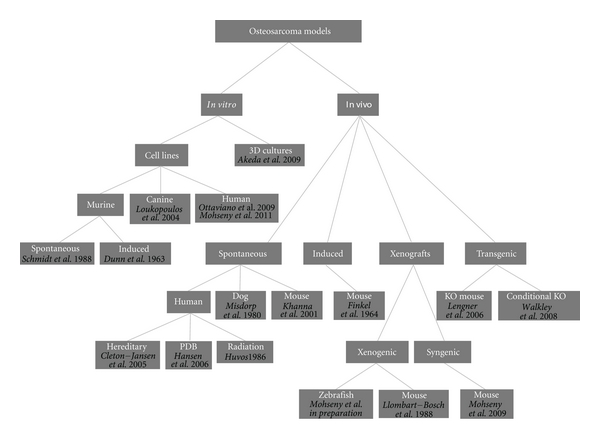
*Osteosarcoma models. *Dendrogram shows reported osteosarcoma models divided into categories. References are provided for each model.

For such a tumor with unknown origin, chaotic genetics, early onset, and aggressive behavior, there is a need for representative models providing possibilities to learn more about the tumor biology in order to find ways to target its cancerous behavior. A similar conclusion was drawn a century ago by Daels and Schurch who induced osteosarcoma in experimental animals by exposing the bones to highly radioactive materials [13–15]. Today an ideal osteosarcoma model representing its biological, genetic and clinical features under practical lab conditions is still missing. Nevertheless, a number of human osteosarcoma cell lines have been established and extensively characterized [16–18], spontaneous osteosarcoma is reported in mice [19–21] and is quite common in dogs [22, 23], secondary osteosarcoma is found in patients receiving radiation, patients of Paget's disease of bone and within syndromes like Li-Fraumeni and hereditary retinoblastoma [2, 24], numerous syngeneic models have been developed, many xenotransplantation studies have been performed, and even in the latest years conditional mouse osteosarcoma models have been established (Figure 1). Thus, theoretically, there should be possibilities to uncover one or more adequate osteosarcoma models for which we here categorize models proposed in the literature and highlight most recent findings towards modeling osteosarcoma.

## 2. *In Vitro*

### 2.1. Animal-Derived Cell Lines

**Figure 2:**
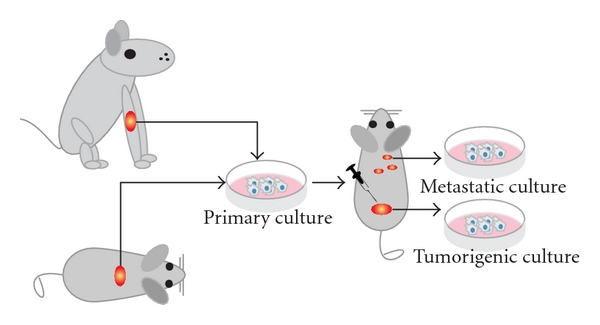
*Animal cell lines. *Schematic representation shows how cell cultures are established from spontaneous canine or murine osteosarcoma. After subsequent injections of the cells into mice, tumorigenic and/or metastatic cultures can be established from the tumors or the metastases, respectively. Please note that dissemination of cells after cell injections is often referred to as metastasis; however, often it is not proved whether these cells are true metastases from a primary tumor or cells that migrated through the body after injection of the cell mass.

The first successfully established metastatic osteosarcoma cell lines were derived from spontaneous mouse osteosarcoma (Figure 2) including cell lines K12 and K7M2 (developed from lung metastases of K7 cells in BALB/c mice) [13, 19, 25]. These lines were successfully proved to be metastatic and in later years used to assay the metastatic process of osteosarcoma [26]. Another murine osteosarcoma cell line, the Dunn cell line, and its derivative cell lines like LM8 were extensively used for screening new osteosarcoma drugs and studying the inhibitory effects of these compounds on angiogenesis and metastasis [27–36].

In contrast to K12 and K7M2 cells, UMR 106-01 was not a result of spontaneous osteosarcoma as it is originating from a ^32^P-induced tumor in a rat [37]. However, because of its *in vitro *and *in vivo *(in mouse tibia) exceptionally phenotypical similarities to human osteosarcoma, and the rapid formation of pulmonary metastasis, this cell line has been studied at length not only in osteosarcoma research [38] but also in several other bone-related studies [38–41].

A number of cell lines have been derived from spontaneous canine osteosarcoma (Figure 2). In particular, D-17 cell line originating from an osteosarcoma metastatic to the lung in an 11-year-old female poodle [42] was next to extensive use in viral studies, helpful for analyzing immunotherapy of hepatic micrometastases [43] as well as finding therapeutics for the treatment of bone cancer in dogs.

### 2.2. Human-Derived Cell Lines

**Figure 3:**
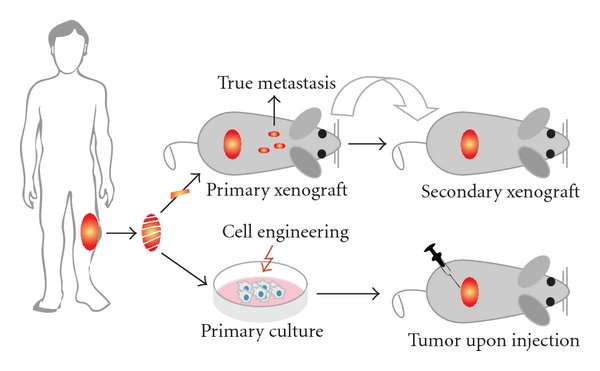
*Human cell lines and xenografts*. Schematic representation of how xenografts and cell cultures are established from human osteosarcoma. After excision, the tumor is dissected into pieces of which one ore more can be subcutaneously—or when technically possible orthotopically—xenotransplanted into nude mice. Please note that when dissemination is found in this situation, these cells should be originating from the primary tumor and more closely representing true metastasis. Subsequently, the process of xenotransplantation can be repeated as often as needed to keep the human tumor “alive” providing a source for research material. Alternatively, after tumor excision, cell cultures can be established. Subsequently, cells can be engineered—like the human osteosarcoma cell line HOS—and injected into mice to assay the tumorigenicity, metastatic potential, and other features. When established xenografts and cell lines are shown to represent certain aspects of human osteosarcoma, they can be used as models.

**Figure 4:**
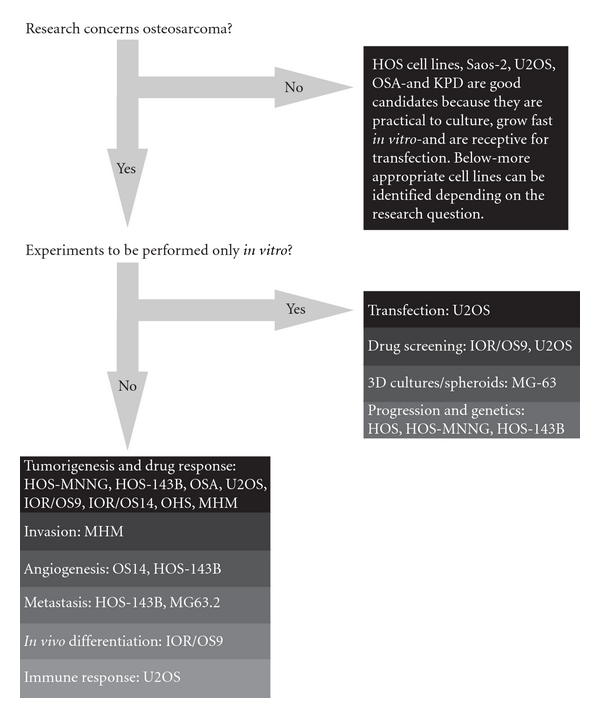
*Human osteosarcoma cell lines*: *which cell line to use?* The flow diagram suggests appropriate cell lines to use for certain research question. Decision-making for this diagram is based on reported genetic, phenotypical and functional analyses of these cell lines, and lab experience from unrelated experiments by the authors and direct collaborators.

Starting with the establishment of the first human osteosarcoma cell line named U2OS in 1964, many human osteosarcoma cell lines, such as HOS and SAOS2, have been successfully established (Figure 3). Despite comprehensive studies characterizing the U2OS cell line *in vitro* [44–46], this cell line could not satisfy the need for an *in vivo* metastatic model [47]. Unfortunately for all subsequent human osteosarcoma cell lines this fact seemed to be an important limitation. Only after characterization of the HOS cell line [48] and production of its many derivative cell lines after genetic alteration [49–53], the first *in vivo *tumorigenic and metastatic cell line, KRIB, later named 143B, was generated [54]. Thereafter, many cell lines were established, described *in vitro *and formed the basis for studying numerous cellular processes either related to cancer or not. Recently (epi)genetic, functional, and *in vivo *characterizations of these cell lines were published to map genetic [16] and epigenetic changes—by expression, methylation, and micro-RNA profiling—differentiation capacity and growth, invasion, and migration potential in nude mice [17]. Based on these reports, together with a previous study of drug resistance in osteosarcoma cell lines [55], appropriate cell lines can be selected for the study of interest (Figure 4).

## 3. *In Vivo*

### 3.1. Cell Lines in Mice

Because of the high frequency of primary tumor development and frequent pulmonary metastases when compared to human osteosarcoma cell lines [13], most often murine osteosarcoma derived cell lines have been inoculated into mice to establish *in vivo *models. Despite the advantage that in these syngeneic models the murine cells can grow in a murine microenvironment, these models do not contain anything of human origin. For this, human osteosarcoma cell lines have been engineered, like HOS virally transformed to 143B and chemically to MNNG, making them successfully tumorigenic and metastatic *in vivo* and useful in animal models [56–59], keeping in mind that a transformed cell line might not be a true representation of the human disease. Nevertheless, these mouse models proved to be excellent models for identifying factors involved in osteosarcoma migration and more importantly for screening drugs to inhibit this [19, 25, 26, 60]. The limitation of such models comes with the fact that studying fully developed osteosarcoma cells does not provide constructive information about the genesis of the tumor and its etiology. In line with this, we recently showed that candidate progenitor mesenchymal stem cells can be used to model osteosarcoma origin and first important events underlying its initiation [61–63]. 

## 4. Xenotransplantation

An alternative to cell injections into recipient animals is to transplant them with a piece of the tumor directly after excision from the patient (Figure 3). In this way, the tumor cells can grow in their own stroma, which is shown to be essential for the tumor behavior [64, 65], and the cells do not need to be cultured *in vitro* lowering the occurrence of additional *ex vivo* changes. Moreover, reports suggest that such xenotransplant models adequately represent the human osteosarcoma characteristics [66, 67] (Kuijjer et al. BMC Cancer, in press). Therefore, Professor Llombart-Bosch and colleagues have xenotransplanted more than 500 sarcomas in mice, kept the tumors “alive” for many years, and studied them through many generations [68–71]. A drawback of this system is that the implant inside the mouse rapidly will be infiltrated by mouse cells possibly influencing the activities of the tumor cells and more importantly if the xenotransplant is excised for cell culturing, the mouse cells can easily overgrow the human cell population (unpublished data). 

## 5. Radio-Carcinogen-Induced Mouse Osteosarcoma

**Figure 5:**
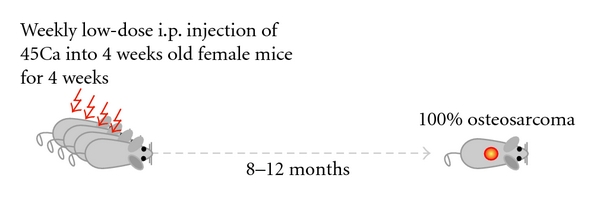
*The radiocarcinogenic model*. Schematic representation of the radiocarcinogenic osteosarcoma mouse model showing that 4-week old mice weekly receive low-dose injections of 45Ca intraperitoneally (i.p.) for four weeks. Within 8–12 months, all female mice develop osteosarcomas. Most lesions are located in the spines of the animals, and metastases are not frequently found. Please note that this figure is based on oral communication with Professor Thomas.

In the sixties, Finkel et al. assayed the influence and parameters of radiation on bone tumor development in mice [72]. By intravenous injections of the bone-seeking radionuclide Ca^45^ in mice, a new osteosarcoma model was born. Although to our knowledge, in the years after, the model has not been reported in osteosarcoma research, recently Professor Thomas and colleagues have reestablished the model (Figure 5). A drawback of the model is that—based on the locations and the histology of the tumors—it most probably and logically represents secondary osteosarcoma as seen in postradiation osteosarcoma patients, which might not share molecular mechanisms with primary osteosarcoma as most often found in young patients with no history of radiation. Nevertheless, as 100% of the female mice develop osteosarcomas within 8–12 months after treatment, the model provides unique opportunities to study this cancer. Moreover, after the formation of the tumors, the model allows for subsequent syngeneic transplantations in immune-competent mice to study the host immune response.

## 6. Canine Osteosarcoma

Spontaneous osteosarcoma is less rare in large dogs than in humans making the dog an attractive candidate model to study the human disease [73]. Moreover, limited genetic variation in inbred dogs allows for statistically acceptable studies even with low number of cases [22]. Although osteosarcoma develops at later age in dogs, its biologic behavior and clinical representation is similar to that of human osteosarcoma [74]. Lately a new canine osteosarcoma model was established in particular for the study of the p53 pathway. In this paper, the authors propose that canine p53 family proteins have biological activities comparable to human equivalents making the dog an excellent outbred spontaneous osteosarcoma model. An idea shared by researchers like Professor Kirpensteijn, who has studied canine osteosarcoma for a long time [22, 74–77]. Despite the availability of this relatively good representative model, major breakthroughs still have not been made in osteosarcoma understanding and treatment for the past 30 years, which might be due to the very same sudden character of the model. Having a spontaneous animal osteosarcoma without any external manipulation and within its natural environment might be the best option mirroring the human situation, but simultaneously it might be as complicated as human osteosarcoma to understand. One should realize that the more representative a model becomes, resembling the real human conditions, the more complicated it will be to understand. The challenge is to find a good balance between the representativity and the controllability of a model.

## 7. Paget's Disease of Bone

Two important factors complicating osteosarcoma understanding are its low prevalence, which makes association and linkage studies difficult, and its sudden onset, which obscures possible premalignant events or lesions. One possibility to come around these problems is to study a bone metabolic disorder called the Paget's disease of bone (PDB) or *osteitis deformans*. With a 0.15–0.95% of osteosarcoma prevalence in PDB patients—a thousand fold higher chance as compared to the normal population—PDB osteosarcoma could be used for genetic studies to find common variations in an uncommon disease [78]. Moreover, as the PDB osteosarcoma is secondary to an underlying predisposing condition, that is, unbalanced bone metabolism, and as it occurs much later in life [24, 79], it provides the opportunity to understand molecular defects involved in osteosarcoma genesis. Accordingly key players of the NF-*κ*B pathway like the *TNFRSF11A* gene could be studied in osteosarcoma. Unfortunately, exactly the opposite might be true as well since secondary osteosarcoma might have a totally diverse biology compared to primary osteosarcoma concordant with its different clinical features like the later age of onset and the location of the tumors. This might explain why PDB osteosarcoma has not often been used as a model to study osteosarcoma despite its possible benefits.

## 8. Genetic Disorders

Although genetic predisposition is only found in few osteosarcoma cases, investigations in syndromes like Li-Fraumeni (*TP53)*, Retinoblastoma *(RB1)*, and DNA helicase-related conditions Rothmund-Thomson (*RECQL4*) and Werner (*RECQL2*) have led to speculation and identification of some of the most important genes involved in osteosarcoma, like the *RB1* gene mutations and *TP53* germline mutations [80, 81]. A big pitfall in using these hereditary syndromes to extract knowledge of osteosarcoma is that they are caused by germline mutations in most important tumor suppressor genes and/or oncogenes resulting in a broad spectrum of malignancies of which osteosarcoma is only a small part. Therefore, any finding from these syndrome related osteosarcoma models might be rather cancer-specific than osteosarcoma specific.

## 9. Highlights

### 9.1. Conditional Mouse Model

Recently, Walkley et al. developed an osteosarcoma conditional mouse model that mimics human osteosarcoma phenotypically and genetically far better than any mouse model established before [82]. Previously a number of mouse strains were reported to develop osteosarcoma upon genetic manipulations, like *TP53* germline mutation [83–85], osteoblast-restricted deletion of *TP53* [86], overexpression of *c-Fos* [87–89], and heterozygous mutation of *Nf2* [90, 91]. However, the long latency of osteosarcoma development combined with the low penetrance and unspecific tumor development made them impractical models. Making use of floxed conditional alleles of both *p53* and *pRb *genes, the Walkley model allows for periodic and tissue-restricted inactivation of these genes resulting into 100% specific and 100% penetrance osteosarcoma development within three to five months. In line with the human osteosarcoma, a substantial number of the *p53^fl/fl^-pRb^fl/fl^*-osteosarcoma-bearing mice developed metastases and high conservation of the genetical changes between mouse and human osteosarcoma was demonstrated. Drawbacks of this osteosarcoma model include the inconsistency in tumor location since human osteosarcoma is mostly located at the ends of the long bones, while in Walkley mice the most frequent affected sites were the jaw and the head. Essential differences between endochondral ossification—formed from cartilage and mainly found in long bones—and intramembranous ossification—formed from connective tissues like the mesenchyme and mainly found in flat bones—might be involved in this discrepancy. More importantly, in this model most metastases were found in the livers of the mice, again not representative of the predominant lung metastases found in human osteosarcoma. Interestingly in secondary osteosarcoma as, for example, PDB osteosarcoma a similar pattern of tumor distribution is more common. Furthermore, the loss of *p53 *and *pRb* in this model might represent osteosarcoma in a genetic predisposing condition parallel to the human situation in Li-Fraumeni and Retinoblastoma patients. The other drawback of the model is its assumption that osteosarcoma is derived from a late preosteoblastic stage (when osterix is expressed), while it is not excluded that the transformation might even happen before this stage [92]. Indeed this might explain the narrow histological spectrum of these mice osteosarcoma showing osteoblastic differentiation as compared to the human osteosarcoma where many subtypes are found [1]. Recent reports show malignant transformation of normal MSCs, which formed osteosarcoma lesions after this transformation [62, 63, 92] providing evidence for an MSC origin of osteosarcoma. Although technically more difficult to hit specifically and conditionally only mesenchymal stem cells, inactivating *p53* and *pRb* in these cells might result in a more representative osteosarcoma model.

### 9.2. Zebrafish Progression Model

**Figure 6:**
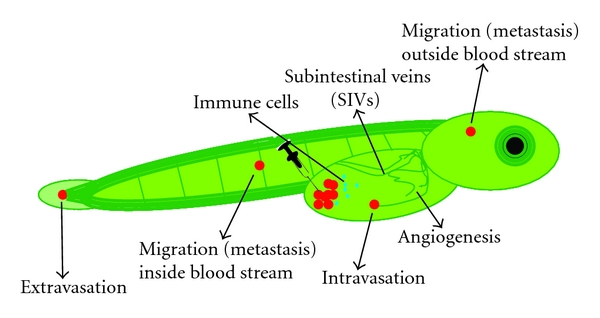
Cell migration, angiogenesis, and host response. Schematic representation of a transgenic zebrafish embryo in which all blood vessels are labeled in green. After injection of cells labeled in red, the transparent embryos are screened daily to investigate processes like intra/extravasation, migration (metastasis), and signs of angiogenesis. In addition, by labeling immune cells with another color (blue in this figure) or by examining the zebrafish gene expression profiles, the host response to the injected cells can be studied.

In the most recent efforts, we have been working towards modeling other aspects of osteosarcoma, which are its highly aggressive local growth and its progression in terms of invasion, angiogenesis, and metastasis. A better knowledge of these processes is essential for designing new effective therapies since the major cause of mortality in osteosarcoma patients is metastasis and not the primary tumor. As indicated, several mouse models are available which could be used to assay these processes; however, next to the above-mentioned drawbacks, they are in general expensive and need a long period of time for tumor initiation, tumor progression, and treatment response. Therefore, a zebrafish embryo model is an attractive alternative in which within only 3 days after tumor grafting, tumor cell homing, proliferation, migration, and angiogenesis (Figure 6, Mohseny et al., manuscript submitted) can be seen. As a proof of principal for the validity of the model, we compared the behavior of the previously published murine MSCs [62] before and after transformation of the cells. Transgenic zebrafish expressing enhanced green fluorescent protein (EGFP) in all blood vessels [93] and transparent zebrafish called Casper [94] were crossed to produce transparent embryos with green blood vessels in which cells with a red label were injected. In contrast to the low passage MSCs, the transformed MSCs showed—within 3 days after injection—excessive proliferation, migration towards the body of the fish, and angiogenesis. The advantages of this model as compared to mouse models are its relatively low costs, its high statistical power by the large group sizes, its high speed—most experiments are done within 5 days—its reproducibility and ease in dosing drugs since these are added into the swimming water of the fish instead of adding to the food, and its advantages in imaging by using transgenic fish. A major drawback of the model is directly related to one of its advantages, that is, the high speed. In its current form—only three to five days of experiment and inside embryos—there is no true tumor and stroma formation lacking a representative microenvironment as compared to the human situation. Nevertheless, with the many advantages and its reported use in other cancers [93, 94], this model provides new opportunities to be exploited in future osteosarcoma studies.

## 10. Discussion

The early onset, lack of a predisposing condition or precursor lesion, and only infrequent (<2%) prevalence of inheritance make osteosarcoma exceptional as compared to other malignancies that result from a multistep genetic process over many years like colorectal cancer. These factors together with the extensive genetic instability of osteosarcoma hamper the elucidation of the tumor's biology [95]. Ideally, a good osteosarcoma model should be able to recapitulate crucial aspects of the human tumor, including osteoid production, expression of biomarkers such as alkaline phosphatase (ALP), osteocalcin (OCN), and osteopontin (OP), rapid tumour growth, local aggressiveness, pulmonary metastases, and the genetic events underlying these processes [59]. However, it is becoming increasingly more evident that creating such a model for osteosarcoma is unrealistic because of a chicken-and-egg situation—models are needed to understand the complex etiology of the tumor while understanding the etiology is needed to set up appropriate models—and so on. To overcome this problem, instead of trying to find a single “perfect” osteosarcoma model, it is more sensible to dissect this extremely complex tumor into parts and to study them separately using adequate models. In line with this, we distinguish four essential aspects in osteosarcoma disease including the sudden rapid and early onset, the high genomic instability, the local aggressiveness and metastasis potential, and the need for targeted therapy. For each of these aspects, there are now suitable models available which could be utilized in the following Sections.

*Challenges in the study of human conventional osteosarcoma:*

- early sudden onset,
- infrequent prevalence,
- no predisposing condition,
- no precursor lesions,
- mainly sporadic,
- extensive genomic instability,
- many genetic alterations,
- wide histological heterogeneity,
- chemotherapy before surgery.

### 10.1. Genesis

To study the early steps in osteosarcoma-genesis, the mouse mesenchymal stem cells (mMSCs) based model—by which substantial evidence for a MSC origin of osteosarcoma was provided [62, 63]—is useful. The clinical validity of the model was confirmed by the finding that loss of *CDKN2A/p16*—one of the critical steps during the malignant transformation of the MSCs—in human osteosarcoma significantly correlated with patients' survival [62]. Moreover, this loss was based on the same molecular mechanism as in the model that is, genomic deletion [61]. For further knowledge of osteosarcoma-genesis, this model provides the possibility to gradually analyze stages of osteosarcoma prior to the fully developed tumor. By comparing transformed MSCs to their normal parental MSCs—preferentially by sequencing assays—driver mutations underlying tumorigenic transformation can be identified as all passenger mutations than would be excluded.

### 10.2. Etiology

As seen in clinical samples as well as in human- or mouse-derived osteosarcoma cell lines, fully developed osteosarcoma is characterized by a high degree of genomic alterations and a continuous state of genomic instability. Although also the above-mentioned mMSC model demonstrated genomic instability of the transformed MSCs and despite the ongoing debate in the literature, solid reports showing transformation of human MSCs to osteosarcoma cells accompanied by genomic instability are still missing. Nevertheless, this disturbance in the integrity of osteosarcoma's genome remains a crucial step in osteosarcoma-genesis and might be studied in two steps. First, more insight into mechanisms involved in genomic instability of mesenchymal cells is needed. A recently published mouse model showing a crucial role of the *Fzr1* gene in maintaining genomic stability [96] provides opportunities to tackle this problem. Bone-marrow-derived MSCs from *Fzr1^-/fl^* mice could be studied to investigate the relation between genomic instability and malignant transformation. Second, since human MSCs do not seem to transform spontaneously, genomic instability can be introduced to them by drugs like nocodazole [97]. Hypothetically as a result of the genomic instability, human MSCs will undergo apoptosis which would require inactivation of the apoptotic pathway, that is, deletion of the *TP53 *or the *CDKN2A* genes, to overcome all the checkpoints and to allow the MSCs to proliferate with abnormal genomes. These cells would provide essential models for the early steps of osteosarcoma genesis.

### 10.3. Local Invasion and Dissemination

To study the aggressive behavior and progression of osteosarcoma in a high throughput manner, the recently established zebrafish model provides a better understanding of osteosarcoma cell invasion, migration, and processes involving angiogenesis. The advantages of this model as compared to mouse models are its relatively low costs, its high statistical power and reproducibility by the large group sizes, its short experimental time span, its ease in dosing drugs since these are added into the water the fish are swimming in instead of adding to the food, and its advantages in imaging by using transgenic fish. Hundreds of parameters can be screened, which would allow for the selection of candidate genes involved in migration and angiogenesis. Moreover, next to the cell characteristics, the host (innate) immune response towards the tumor cells can be monitored. A disadvantage of the model accompanied by the short duration of the experiments is the lack of tumor mass formation. Therefore, all selected cell intrinsic candidate genes and immune-related response genes can be further analyzed in the radiocarcinogenic osteosarcoma mouse model. Currently, Professor Thomas is studying the role of a broad spectrum of immune deficiencies and osteosarcoma formation and progression by using this mouse model (presented at EuroBoNet annual meeting 2011).

### 10.4. Drug Screening

When the acquired basic understanding by the first two steps and the (pre-)screens by the third step successfully identify clinically relevant candidate targets to treat osteosarcoma, models are needed for drug screens. As (pre-) screens again, the zebrafish model provides an efficient manner to select candidate drugs. This time not only tumor cell injections could be used, but also human osteosarcoma-derived micro xenotransplants in the embryos preserving the tumor micro environment. Subsequently, the candidate drugs could be tested in mouse models. One convenient model was identified by *in vivo* characterization of 19 osteosarcoma cell lines showing that the HOS-143B cell line—after subcutaneous injections into nude mice—rapidly produced tumors which metastasized to the lungs. Subsequently, the human osteosarcoma xenotransplanted mice could be used in which the tumor stroma is maintained. Finally, when a model system is needed with an intact immune system, the radiocarcinogenic osteosarcoma model and spontaneous canine osteosarcoma models are most appropriate.

**Solutions to make the study of osteosarcoma possible are presented**

Multiple models can be used to investigate aspects of osteosarcoma:

- initiation → mMSCs mouse model,
- genomic instability →*Fzr1* mouse model,
- invasion, dissemination, and host response → zebrafish cell injection and xenotransplantation models,
- drug screening → Zebrafish, (radiocarcinogenic) mouse and canine models.

Conventional chemotherapy has been essential to improve the survival of osteosarcoma patients up to 60–70% but has reached a plateau phase. Attempts to invent more effective chemotherapeutic regimens have failed to further improve survival [8]. Therefore, specific targets identified and developed by comprehensive basic research and wide screens of available drugs—the rarity of osteosarcoma hampers development of new compounds by pharmaceutical companies—are needed to precede clinical trials.
